# Supplementary figures and images for: Biological complexity facilitates tuning of the neuronal parameter space
Source: PLoS Comput Biol. 2023 Jul 3;19(7):e1011212. doi: 10.1371/journal.pcbi.1011212 (PMC10353791; doi:10.1371/journal.pcbi.1011212)

**A**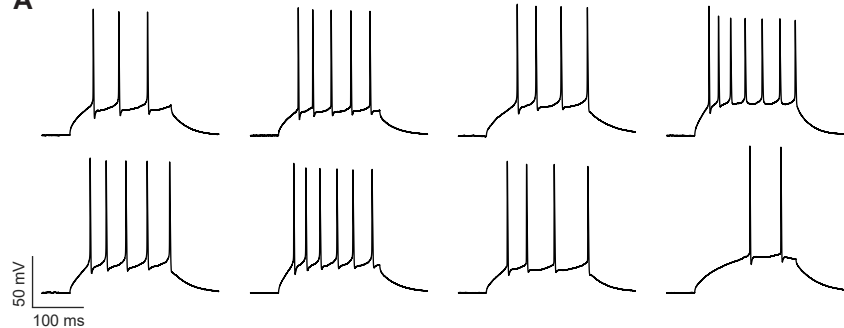**B**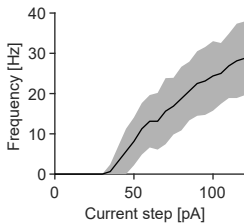**C**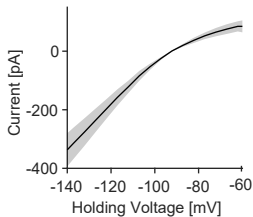**D**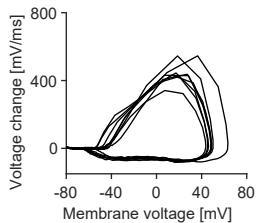

Supplement: S1 Fig — Experimental data from [30]. A, Voltage traces of eight different GCs during 200ms current clamp injection of 90pA. B, Frequency of action potentials elicited by 200ms lasting current injections (mean and standard deviation from raw traces, experimental standard deviation is shown as grey patches). C, Current-voltage (I–V) relationships (mean and standard deviation from raw traces, experimental standard deviation is shown as grey patches). D, Phase plots of the first action potential during 90pA current clamp. Modified from Fig 2 in [23]. (PDF) [file pcbi.1011212.s001.pdf]

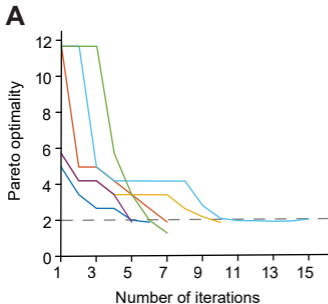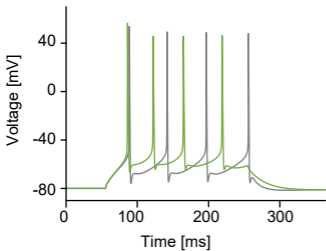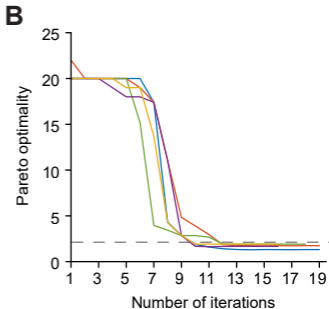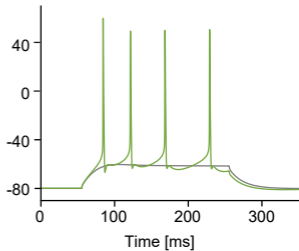

Supplement: S2 Fig — A, Temporal evolution of Pareto optimality (top, see Eq 4) using the gradient descent method. Solutions are considered valid once their Pareto optimality drops below 2 (dashed line). Initial parameter combinations are random non-valid parameter combinations within a range between 0× and 2× the value in the reference parameter set. (bottom) Voltage traces of the model with initial parameter combinations (grey) and optimised parameters (green). B, Same as in A, but all initial parameter combinations were in a similar order of magnitude of Pareto optimality with corresponding models that did not even produce spikes. (PDF) [file pcbi.1011212.s002.pdf]

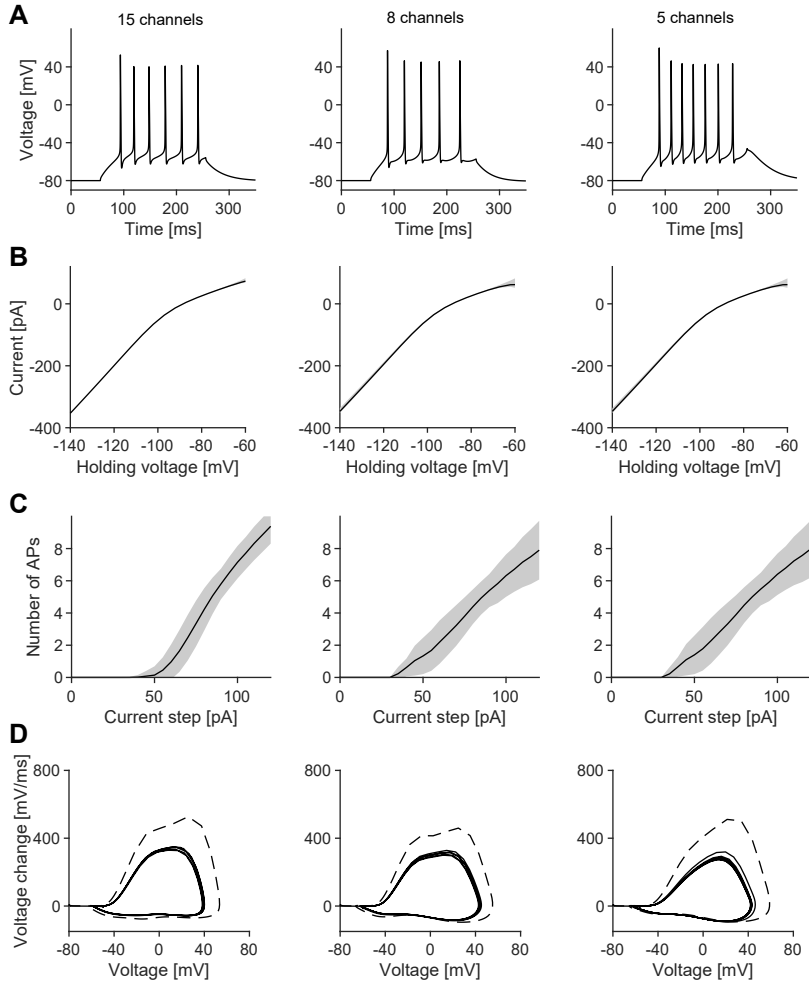

Supplement: S3 Fig — A–D, Similar panels as in S1 Fig for the different models and respective parameter combinations as in Fig 2A. (PDF) [file pcbi.1011212.s003.pdf]

15 channels - BK

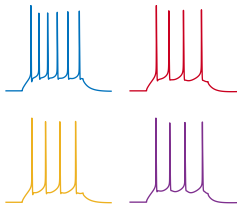

15 channels - Cav22

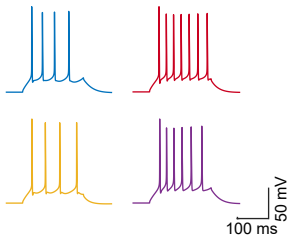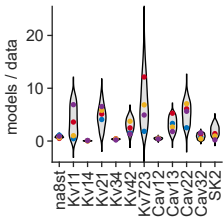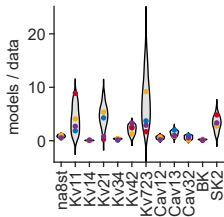

Supplement: S4 Fig — Valid parameter combinations in the fully complex model are well spread and more stable as compared to reduced models. Activity traces of 4 randomly picked valid parameter combinations in models successfully compensating the corresponding knock-out (Top). Coloured dots illustrate conductance densities of the four valid parameter combinations shown in top traces (Bottom). Violin plots show the probability distribution of valid parameter combinations. Conductances are weighted by the surface area of the corresponding membrane regions. (PDF) [file pcbi.1011212.s004.pdf]

**A**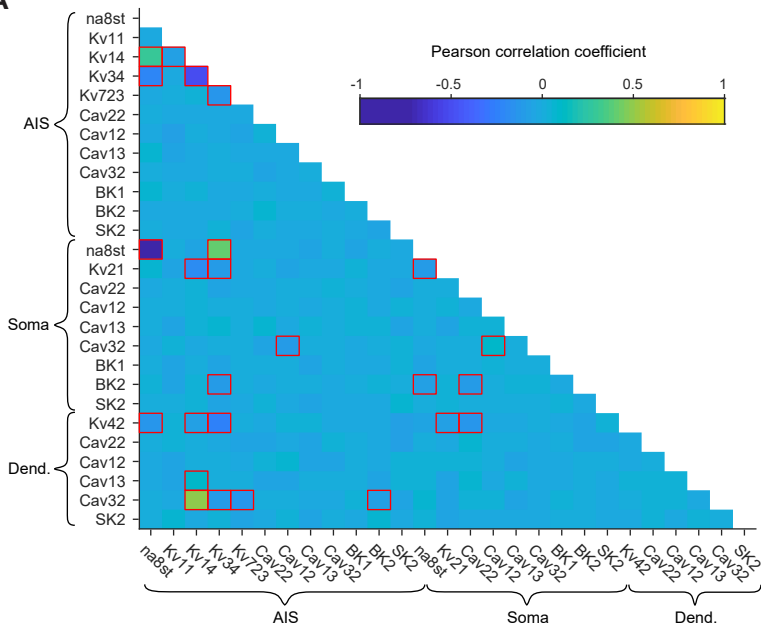**B**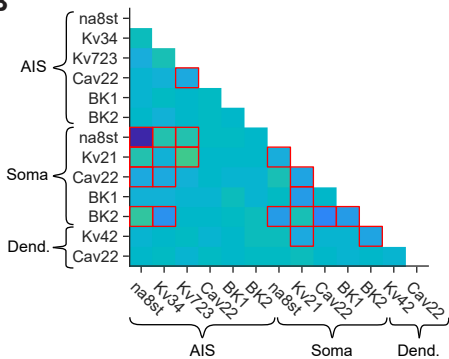**C**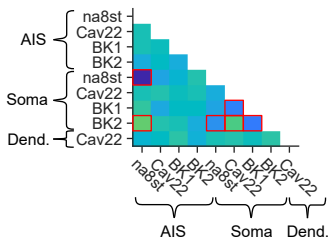

Supplement: S5 Fig — Significant correlations are highlighted by red boxes (p-value <0.01). Pairwise correlations in population of A, 15−channel models, B, 9−channel models, C, 5−channel models. (PDF) [file pcbi.1011212.s005.pdf]

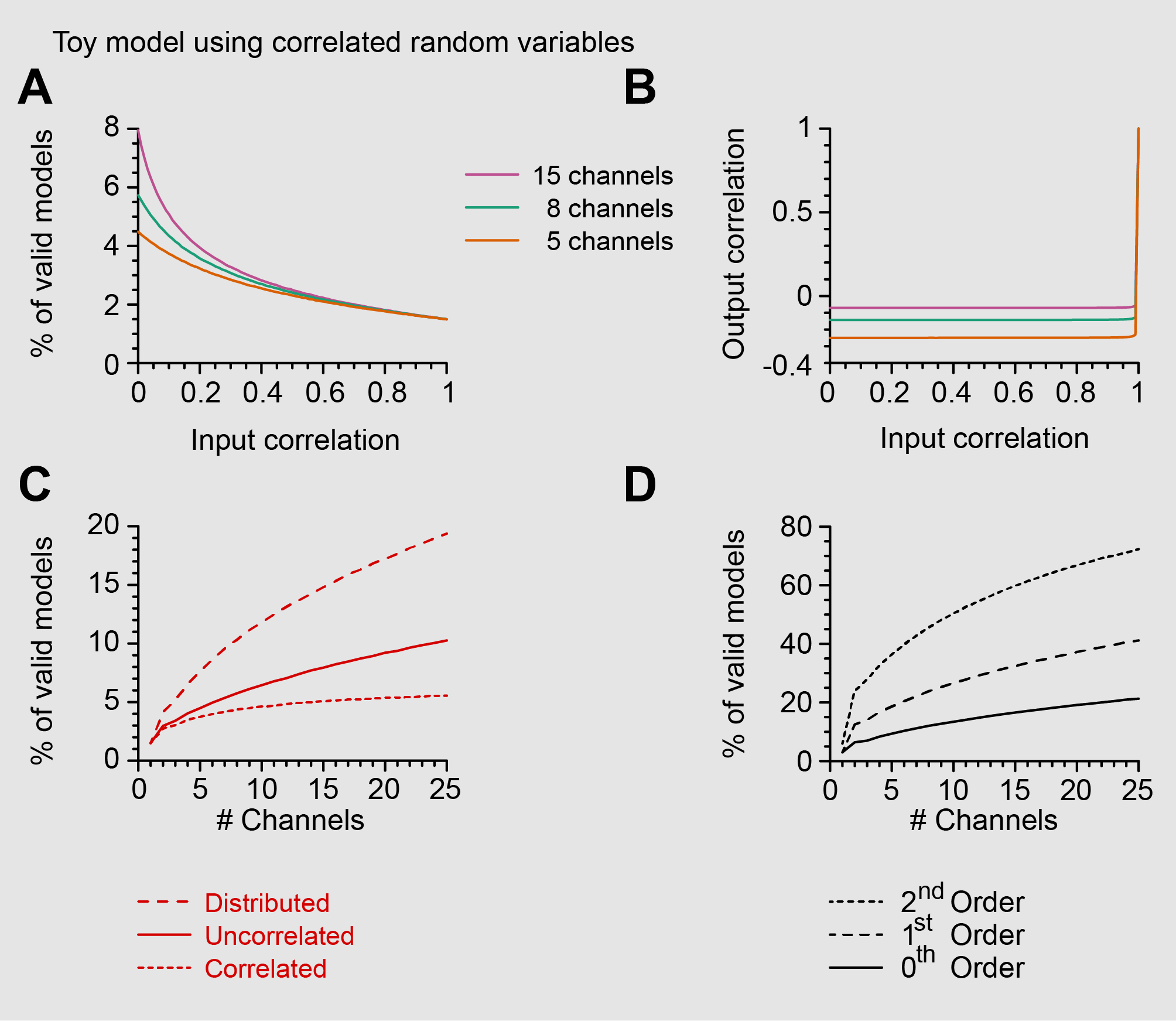

Supplement: S6 Fig — A, Effects of pairwise correlations on the proportion of valid models for different numbers of variables. The 1 variable model is not plotted as it is not affected by correlations. All models converge to the same point as their elements become perfectly correlated and the effective number of dimensions is reduced to 1. B, Observed output correlations in valid models as a function of the pairwise correlation used to generate the population from which valid models are drawn. For almost all input correlations the observed correlation depends only on the number of variables. C, Distributing parameters more evenly in space (dashed line) led to even more solutions than in the independent model (solid line) and the positively correlated model (dotted line). D, Adding hierarchical interactions and nonlinearities improved the validity of the models (Eq 4). v0 (solid), v1 (dashed), and v2 (dotted). (PNG) [file pcbi.1011212.s006.png]

**A**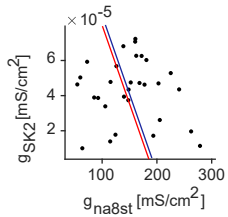**B**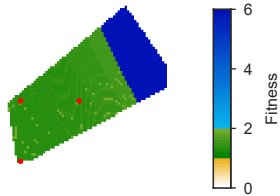**C**

-10%

-5%

+5%

+10%

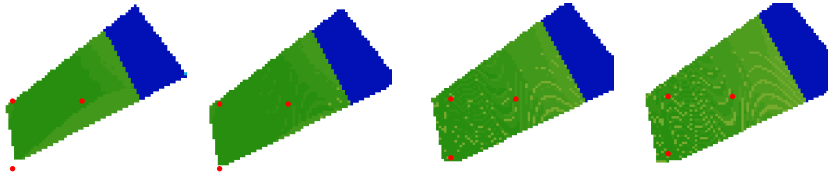

Supplement: S7 Fig — Hyperplane analysis inspired by [15] for the 15−channel model. A, The hyperplane of B is shown in red as projection onto gNa8st,AIS vs. gSK2,AIS plane. 25 randomly chosen valid parameter combinations are represented by dots. The blue hyperplane is parallel to the red and is defined by the addition of 10% of the SD of all solutions (in every dimension). B, Hyperplane defined by the three individuals on the red line in A. The Fitness of all points is colour scaled. The three original individuals are highlighted as red dots. C, The red dots mark the places parallel to the 3 originally selected individuals. (PDF) [file pcbi.1011212.s007.pdf]

**A**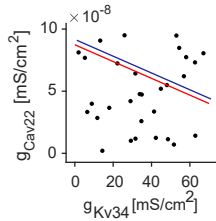**B**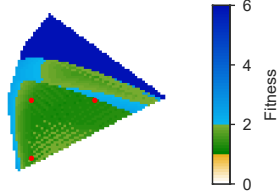**C**

-10%

-5%

+5%

+10%

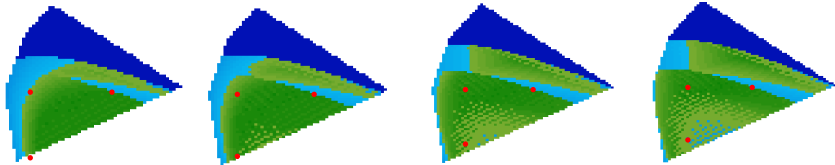

Supplement: S8 Fig — Hyperplane analysis inspired by [15] for the 9−channel model. A, The hyperplane of B is shown in red as projection onto gKv34,AIS vs. gCav22,AIS plane. 25 randomly chosen valid parameter combinations are represented by dots. The blue hyperplane is parallel to the red and is defined by the addition of 10% of the SD of all solutions (in every dimension). B, Hyperplane defined by the three individuals on the red line in A. The Fitness of all points is colour scaled. The three original individuals are highlighted as red dots. C, The red dots mark the places parallel to the 3 originally selected individuals. (PDF) [file pcbi.1011212.s008.pdf]

**A**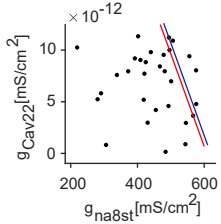**B**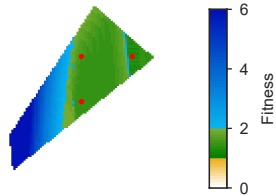**C**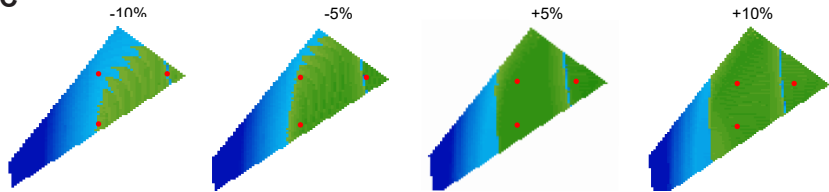

Supplement: S9 Fig — Hyperplane analysis inspired by [15] for the 5−channel model. A, The hyperplane of B is shown in red as projection onto gna8st,AIS vs. gCav22,AIS plane. 25 randomly chosen valid parameter combinations are represented by dots. The blue hyperplane is parallel to the red and is defined by the addition of 10% of the SD of all solutions (in every dimension). B, Hyperplane defined by the three individuals on the red line in A. The Fitness of all points is colour scaled. The three original individuals are highlighted as red dots. C, The red dots mark the places parallel to the 3 originally selected individuals. (PDF) [file pcbi.1011212.s009.pdf]
