# Supplementary material for: Biological complexity facilitates tuning of the neuronal parameter space
Source: PLoS Comput Biol. 2023 Jul 3;19(7):e1011212. doi: 10.1371/journal.pcbi.1011212 (PMC10353791; doi:10.1371/journal.pcbi.1011212)
Supplement: S1 Table — Ion channels and their expression profiles in the corresponding morphological compartments. Conductance densities are given in units of mScm2. (PDF) [file pcbi.1011212.s010.pdf]

Name	AIS	Soma	Dendrite
pas	6.593×10^{-6}	1.385×10^{-5}	1.385×10^{-5}
Kir 2.1	6.741×10^{-5}	1.415×10^{-4}	1.415×10^{-4}
Na8st	0.614	0.1478	
Kv 1.1	2.76×10^{-4}		
Kv 1.4	1.77×10^{-2}		
Kv 2.1		0.0022	
Kv 3.4	0.6987		
Kv 4.2			0.0039
Kv 7.2/3	0.0031		
Cav 1.2	3.1×10^{-4}	7.1×10^{-5}	2×10^{-5}
Cav 1.3	5.48×10^{-6}	2.5×10^{-5}	3.7×10^{-6}
Cav 2.2	3.19×10^{-7}	7.4×10^{-5}	5.8×10^{-6}
Cav 3.2	1.22×10^{-5}	1.6×10^{-5}	3.8×10^{-5}
BK			
α	0.0018	9.3×10^{-4}	
β	0.51	0.0148	
SK2	1.1×10^{-5}	3.7×10^{-8}	8.5×10^{-7}
