# Supplementary material for: Biological complexity facilitates tuning of the neuronal parameter space
Source: PLoS Comput Biol. 2023 Jul 3;19(7):e1011212. doi: 10.1371/journal.pcbi.1011212 (PMC10353791; doi:10.1371/journal.pcbi.1011212)
Supplement: S2 Table — Ion channels and their expression profiles in the corresponding morphological compartments. Conductance densities are given in units of mScm2. (PDF) [file pcbi.1011212.s011.pdf]

Name	AIS	Soma	Dendrite
pas	6.593×10^{-6}	1.385×10^{-5}	1.385×10^{-5}
Kir 2.1	6.741×10^{-5}	1.415×10^{-4}	1.415×10^{-4}
Na8st	0.4925	0.0881	
Kv 2.1		0.0071	
Kv 3.4	0.0339		
Kv 7.2/3	0.0074		
Kv 4.2			0.0022
Cav 2.2	4.77×10^{-11}	4.5×10^{-4}	3.56×10^{-5}
BK			
α	1.25×10^{-7}	0.0043	
β	0.0148	0.0156	
