# Supplementary material for: Biological complexity facilitates tuning of the neuronal parameter space
Source: PLoS Comput Biol. 2023 Jul 3;19(7):e1011212. doi: 10.1371/journal.pcbi.1011212 (PMC10353791; doi:10.1371/journal.pcbi.1011212)
Supplement: S3 Table — Ion channels and their expression profiles in the corresponding morphological compartments. Conductance densities are given in units of mScm2. (PDF) [file pcbi.1011212.s012.pdf]

Name	AIS	Soma	Dendrite
pas	6.593×10^{-6}	1.385×10^{-5}	1.385×10^{-5}
Kir 2.1	6.741×10^{-5}	1.415×10^{-4}	1.415×10^{-4}
Na8st	0.306	0.119	
Cav 2.2	5.82×10^{-15}	8.64×10^{-4}	1.22×10^{-4}
BK			
α	1.16×10^{-7}	0.0132	
β	1.321	0.0185	
