# Supplementary material for: Biological complexity facilitates tuning of the neuronal parameter space
Source: PLoS Comput Biol. 2023 Jul 3;19(7):e1011212. doi: 10.1371/journal.pcbi.1011212 (PMC10353791; doi:10.1371/journal.pcbi.1011212)
Supplement: Attachment — Submitted filename: response.pdf [file pcbi.1011212.s013.pdf]

Bernstein Preis
Computational Neuroscience
Hermann Cuntz

Ernst Strüngmann Institute
in Cooperation with Max Planck Society

FIAS Frankfurt Institute
for Advanced Studies

To: **PLOS,**
1265 Battery Street, Suite 200,
San Francisco, CA 94111, USA

May 3, 2023

Dear editors of *PLoS Computational Biology*,

Thank you very much for your comments and suggestions regarding our manuscript entitled '**Biological complexity facilitates tuning of the neuronal parameter space**', which we had submitted to *PLOS Computational Biology*.

We are grateful to the reviewers for their suggestions to improve the manuscript. We have revised the manuscript according to the points raised by the reviewers. Please see a detailed point-by-point response below.

Please do not hesitate to contact us if you have further questions; we look forward to hearing from you,

Marius Schneider, Alexander Bird, Albert Gidon,

Jochen Triesch, Peter Jedlicka, Hermann Cuntz

Marius Schneider
Ernst Strüngmann Institute (ESI) for Neuroscience in Cooperation with Max Planck Society
Deutschordenstraße 46, D-60528 Frankfurt am Main

Responses to the comments of the editor and the reviewers

For the details of our revision please refer to the point-by-point answers to reviewer comments provided below.

Reviewer # 1

Overview: This is an important paper that addresses a fundamental set of questions relevant to the expression of ion channels in neurons. The direct comparisons of the 15 channel conductance model to the 5 conductance model is quite lovely. One of the results I was initially surprised by, but makes sense after thinking, is that the reduced models showed stronger and different pairwise correlations than the full model.

We agree that this is an important part of our results that helps to explain why the space of valid parameters is much more constrained in the reduced model. In the reduced model there are fewer other parameters available to compensate for changes in any single parameter, the strength of any compensations will then likely be stronger and lead to stronger pairwise correlations. This effect is mirrored in the toy model (see below). We have added a brief additional comment in the manuscript to explain why this occurs.

Specific comments

1. Should Swensen (2005) be Swensen and Bean (2005)?

Our response 1: Yes, we changed the reference.

2. Some of the 9 features that were used might be correlated? So for example, firing rate and ISI? Is this important? Does it matter?

Our response 2: Yes, some of the features will be correlated. This does not cause a problem for the optimisation procedure as it always tries to optimise the spiking feature that shows the strongest

deviation from the experimental data. We have added a short note in the results section: "The fact that some of the 9 spike features that we are optimising are correlated (e.g. Firing rate and ISI) did not cause a problem for the optimization procedure as it always tries to optimise the spiking feature that shows the strongest deviation from the experimental data."

3.In my mind, the business with the law of large numbers is just a distraction from the beauty of the results. But I understand that the authors might disagree strongly, and that is fine. I just am asking them to think through this once again.

Our response 3: Thank you for pointing this out. We agree that the direct results are interesting in themselves. Nonetheless, we think that it is best to keep our toy model and its analogy with the law of large numbers since we believe it adds to the understanding of the reasons for our results. We also find the extensions of the toy model to correlated inputs and complex interrelationships to be important as it shows that the law of large numbers applies more generally than might be appreciated. Further, the strength of pairwise correlations in the valid versions of the toy model qualitatively match the strengths of these correlations in the compartmental model, with fewer parameters forcing stronger correlations. The toy model might help the reader understand this important effect better.

4. The first paragraph of the Discussion is just a recap of the results. I understand many people do this, but to me this is already in the Abstract and Results, and doesn't need to be repeated. Instead the first paragraph of the Discussion should set the conceptual stage and framework for the work. This is especially true because the Discussion is already long. Moreover, other parts of the Discussion can probably be shortened a bit, and would enhance the force of the new findings rather than just reiterating them?

Our response 4: To address the concern of the reviewer, we have shortened the first paragraph (l. 346 - 353) and the text on l. 384. However, we also had to extend a few parts of the Discussion to address the concerns of the reviewers. Nevertheless, if the Discussion were to be shortened further, we could remove the "homeostatic" paragraph l.372 to 395 (but we would prefer to keep it).

Reviewer # 2

The paper of Schneider et al. addresses an important question in computational neuroscience, namely what is the best level of model complexity when modeling neurons. In particular, they study whether a 15-channel model of a hippocampal granule cell, with its large number of a priori unknown parameters, nevertheless is preferable to a simple 5-channel model with fewer unknown parameters. The claim is that it is, at least when comparing the fraction of acceptable ("valid") model parameter combinations found from randomly perturbing parameters around a "valid" starting solution.

Our response 5: Thank you for the nice comments. We believe that our insights are not only applicable to modelling. They can also potentially be informative about the underlying biology.

I find the problem and results to be both important and timely, and I find the manuscript highly suited for PLoS Computaional Biology. However, I have a "major" question and some "minor commment/questions" that I would like to see addressed by the authors.

Major:

- On p. 8 it reads: "Since we were starting from a valid parameter combination, we decided to use a gradient descent algorithm (Press et al., 2007) in combination with random parameter space exploration (see Methods)." Using gradient descent seems a bit unsuited to answer how many good solutions there are in the parameter space in general. Instead of seeing how many solutions there are in general, doesn't it rather check how easy it is to find solutions? Couldn't the observation be explained by the gradient descent working better in high-dimensional system since the chances of being stuck in a local minimum is lower (since the chances of being stuck at local error-minimum with a valley in all directions is lower the more dimensions there are). As I understand it, this has been used as an argument for why one often finds a good minimum when fitting deep networks, see Sejnowski et al, PNAS, 2020 (`https://www.pnas.org/doi/10.1073/pnas.1907373117`). By this reasoning it seems that sampling parameter space in a more random way around the "valid solution" is necessary to diambiguate the current interpretation in the paper from the alternative possibility that it is easier to find good solutions using gradient descent in high-dimensional neural models (which also would be an interesting result). I suggest that the authors pursue this.

Our response 6: What Reviewer 2 suggests is actually exactly what we did. We only used the optimization algorithm to find one valid parameter set for every model (15, 9, and 5 channel model). From then on we used random sampling to generate a population of valid models for each of the GC models of different complexity (15, 9, and 5 channel models). We agree that using gradient descent alone would not be well suited to test how many good solutions exist. We believe that our phrasing must have been ambiguous so we decided to change the following description of what we did:
"To find a baseline model with a valid parameter combination that fulfills the cost function, we decided to use successive line minimizations in conjugate directions (Brent, 2013) in combination with random parameter space exploration (see Methods). This method also led to good parameter combinations within a few iteration steps when starting from random parameter sets for which the model deviated from the experimental results. By combining random parameter exploration with successive line minimizations in conjugate directions, parameter combinations could even be found when starting from initial parameter sets for which the models produced no spikes at all (Figure S2). Note that the optimization algorithm was only used to generate the baseline models. The populations of valid GC models were all generated based on the baseline models by uniformly random sampling of the channel densities and subsequent counting of valid models."

Minor points:

- I think the model for completeness should be included

Our response 7: The basis GC model is in principle the one published previously in Beining et al., 2017. In addition, we plan on making all code, all models and all data available on Zenodo upon publication.

- It should be clearly stated somewhere what paramters are allowed to vary (only channel densities I presume?)

Our response 8: We changed the phrasing in the following way:
"In order to check this, we first generated $20,000$ random model instances for each of the three baseline models by uniformly sampling the individual conductance densities within a range between $0\times$ and $2\times$ the value in the baseline model (all channel densities beside pas and Kir 2.1 in Table S1-S3 were sampled)."

- p. 8: I do not see how the referenced figure S1 helps explain the fitness function that was used.

Our response 9: That is correct. Figure S1 shows the electrophysiology traces that were approximated by the fitting procedure and only Figure S3 compares these traces with the model. We changed the wording in the following way:
"To assess the quality of any individual set of parameters more automatically, we designed a fitness function that quantified the distance to experimental spiking data (see Figure S1 for experimental data, Mongiat et al. 2009) and was inspired by approaches used previously (Druckmann et al., 2007; Beining et al., 2017, see Methods)."

- On p. 9 it says ".. a small number of membrane time constants ...". This is confusing: Isn't the membrane time constant defined as the product of the leak membrane resitance and the membrane capactitance?

Our response 10: This was a typo. What we actually meant was time constants in the ion channels. Thank you for catching that. We now write:
"Since GCs have a relatively simple electrophysiological repertoire (nevertheless responsible for sophisticated integration of excitatory and inhibitory information), a small number of ion channel time constants was sufficient to generate adequate firing patterns."

- p. 11: I am not sure I follow why channel independence implies robustness? Maybe expand more on this idea.

Our response 11: Thank you for the comment. We added a few sentences to the reuslts section and hope it now became clearer.
"Any correlation in the parameter space restricts the space of valid solutions to the hyperspace constrained by the channel correlations. This reduces robustness as perturbations from valid solutions that don't follow the constraints given by the channel correlations end up showing non-valid electrophysiological properties. If channels are independent there is more volume in the parameter space that could be occupied by valid solutions. We made similar observations when adding correlations to the parameter space of our toy model (see Results: "Toy model points to law of large numbers")."

- p. 21: Difficult to understand following sentence: "Neurons are thought to target certain desired set points (or

set ranges) in the output space (i.e. function or behaviour space) corresponding to valid points or subspaces in the high-dimensional parameter space of expression levels of ion channels."

Our response 12: Thank you, we have simplified the sentence. Now it reads: "Neurons are thought to target certain desired subspaces in the output space (i.e. function space, e.g. defined by firing properties) corresponding to valid subspaces in the high-dimensional parameter space of ion channel properties."

- p. 21/22: "Our computational analysis indicates that a complex high-dimensional parameter space supports the stability of neuronal excitability against perturbations that would push neurons into non-functional subspaces. The reason is that the topology of the high-dimensional space increases the likelihood of neurons returning into functional subspaces by random ion channel parameter adjustments.". The topology of this high-dimensional space is not known and it is unclear that one can use it as a reason for this behavior. What can probably be claimed is that the volume of the implied fitness functions is larger around solutions in higher dimensions, and volume is not a topological property

Our response 13: We agree that we cannot make statements about the topology of the high-dimensional parameter space. To address this point of the reviewer, we changed the phrasing in the following way:
"Our computational analysis indicates that a complex high-dimensional parameter space supports the stability of neuronal excitability against perturbations that would push neurons into non-functional subspaces. The reason for this is that the volume of valid solutions in the parameter space around a valid parameter set is larger in higher-dimensional parameter spaces than in lower-dimensional ones. This in turn increases the likelihood of neurons returning into functional subspaces by random ion channel parameter adjustments."

Reviewer # 3

The authors use detailed dentate granule cell models to show that having a larger complement of ion channel types increases the robustness of these models to perturbation of channel densities and properties. This is an important point which is demonstrated convincingly in the manuscript. The substance being solid, my comments

and suggestions below are mostly concerned with improving the presentation of these results.

Main point

The main result of Golowasch et al. (2002), a key paper that is cited several times in the present manuscript, is that averaging can fail due to nonlinear interactions between different types of ion channels.
In contrast, you are demonstrating success of averaging, as illustrated by the analogy between the detailed biophysical models and the toy model (= averaging) in the percentage of valid models as a function of the number of variables (Fig. 3).
It therefore seems odd that you are not mentioning or discussing the main result of Golowasch et al. (2002) explicitly. Can you explain what enables the success of averaging in your case, given that Golowasch et al. conclusively demonstrated that averaging of neural models can also fail under certain conditions? Is it simply because Golowasch et al. studied averaging across different neurons/neuron models while you are studying averaging across multiple related channel types within neurons/neuron models? Is it because your baseline models are optimized, highly valid models, and the variability you generate in the population around the baseline models respects the average represented by these valid baseline models by design? Is it because averaging indeed fails 94% to 99% of the time also in your case, if we are using your choice of uniform random sampling in the range of 0x to 2x the baseline conductance, and your definition of a valid model? Are there other possible reasons?

Our response 14: There are significant differences between our toy model and the conductance-based models of Golowasch et al. The most significant is that the 'objective' of the toy model is simply to take a value close to the average of its parameters. This means that, by construction, the 'average' toy model will lie within the set of valid models, whereas Golowasch et al found that their 'average' conductance-based model lay outside the concave set of valid models in their parameter space. In contrast, our conductance-based models would display similar behaviour to Golowasch et al, where averaging parameters over the set of valid models would likely produce an invalid model. Further, we do not produce our populations of valid conductance-based models by averaging parameters, they are produced by randomly drawing points in a high-dimensional box centered at a known valid model. This means that the valid models can lie on any manifold in parameter space (see our Supplemental Figures 7 and 8), and are not forced out of a valid region by the location of the mean as in Golowasch et al. We have now added a brief comparison of our and Golowasch et al.'s results in the Methods, and an explicit description of this difference between the toy and full

models when discussing Figure 3 in the Results. See line 268 and line 651.

Minor points

1. l. 87f "large populations of stochastically generated models (Prinz et al., 2003;": Prinz et al. (2003) introduced the databasing approach, but their database used a regular grid of parameter values, not stochastically generated ones. The references are well chosen but this sentence should be rewritten to reflect the regular grid in Prinz et al. (2003) (see also l. 95f).

Our response 15: We added the reference to another paragraph of the manuscript.
"The model consists of 27 conductance parameters, which precludes a comprehensive grid scan (as in e.g. Prinz et al. 2003) for parameter fitting due to the long computing time in a 27 dimensional parameter space."

2. l. 48ff "it remains unclear what role exactly the diversity of ion channel types plays regarding evolution and its contribution to functional mechanisms that impact neuronal computations.": can you say this in plain English?

Our response 16: The amended sentence now reads: "However, it remains unclear how the diversity of ion channel types is related to the tuning of their parameters in the context of robust and flexible neuronal behaviour."

3. l. 155f "a small number of membrane time constants was sufficient to generate adequate firing patterns.": do you really mean membrane time constants here? Usually, the membrane time constant is understood to be the passive membrane time constant $tau_m = R_m * C_m$.

Our response 17: This was a typo. What we actually meant was time constants in the ion channels. Thank you for catching that.
"Since GCs have a relatively simple electrophysiological repertoire (nevertheless responsible for sophisticated integration of excitatory and inhibitory information), a small number of ion channel time constants was sufficient to generate adequate firing patterns."

4. The last paragraph in the Results section "The GC model cost function", lines 117-128, refers to "The search algorithm", lines 595-619 in Methods. This search algorithm is however used only in two specific instances in Results, namely for finding the parameters of the baseline reduced models in Fig. 1C (see also l. 617), and for finding the parameters of the baseline models with additional channel isoforms in Fig. 4 (l. 323-325; not listed in l. 617). My issue here is solely with the presentation: you should make it clear when the search algorithm is used (i.e. only to generate some baseline models) and when it is not (i.e. for what are arguably the main points of the paper, made using the "database approach" employing uniformly random scaling of channel densities and subsequent counting of valid models in Figures 2, 3A-C and 4 [after the baseline models have been established]). In restructuring those parts of Results that mention the search algorithm, you should aim to cleanly separate your use of the database approach from your use of the search algorithm to avoid confusing the reader.

Our response 18: Thank you for the comment. We hope it now became clearer when we used which approach. See also our response 6.

5a. l. 597f "In order to search for local minima we used a conjugate gradient descent technique (Press et al., 2007).": Please be more specific. Are you in fact not using conjugate gradient methods (which depend on the derivatives of the function to be minimized to be explicitly available) but successive line minimizations in conjugate directions (see also l. 606ff), which works without derivatives being available? Please state the name of the minimization function you used, the source of the implementation, and the original reference for the algorithm (e.g. Brent, R.P. Algorithms for Minimization without Derivatives, Prentice Hall, Englewood Cliffs, NJ, chapter 7.)

Our response 19: Thank you for the comment. Indeed, we used successive line minimizations in conjugate directions. We changed the wording to avoid confusion and added the reference suggested by the reviewer.
See also our response 6.
and
"To search for parameter sets that match our criteria for valid GC activity we combined random sampling with successive line minimizations in conjugate directions (Brent, 2013). Starting from a random or given point in the parameter space, we evaluate the change of the cost function for each dimension with two sample points to smooth the slopes. The algorithm evaluates the fitness function in each dimension and moves in the direction of the steepest descent with respect to the cost function."

5b. How are you dealing with the fact that some of the features in your cost function have discrete, integer values (e.g. 1. numbers of spikes fired within 200 ms under current clamp) while the minimization algorithm you use works best with smooth objective functions whose gradients are also smooth and finite?

Our response 20: Since we combined successive line minimizations in conjugate directions with randomization the fact that our gradients are not smooth did not cause a problem.
We thank the reviewer for the comment and expanded on this issue in the following way: ”For some initial parameter combinations, large areas of the parameter space were completely flat (i.e. the gradient was zero). This was especially the case when the initial models showed no spiking activity (Figure S2B). In this case, we increased the size of the iteration steps consecutively by $\pm$5%. If still (after increasing the step size to $\pm$50%) no gradients other than zero were found or the local minima did not fulfill the criteria of functional GC excitability, we randomised the parameters in the next step in an iteratively increasing range (from $\pm$10% of the corresponding parameter values in steps of $\pm$10% up to $\pm$50%). This approach also helps us with the fact that some spiking features we optimize have discrete integer values (whose gradients are not smooth and finite).”

6a. l. 623f “Each artificial channel form obtained in such a way was associated with a randomised time constant between 0x and 2x the value in the original GC model to obtain altered dynamics.”: was that range sampled uniformly? If yes, please say so.

Our response 21: We thank the reviewer for the comment and changed the wording in the following way:
”Each artificial channel form obtained in this way was assigned a randomised time constant, which was uniformly sampled between $0\times$ and $2\times$ the value in the original GC model, in order to obtain altered dynamics.”

6b. l. 191f “by randomly sampling the individual conductance densities within a range between 0x and 2x the value in the baseline model.”: was the range sampled uniformly?

Our response 22: Yes, the range was sampled uniformly. We changed the phrasing in the following way:
”In order to check this, we first generated $20,000$ random model instances for each of the three baseline models by uniformly sampling the individual conductance densities within a range

between $0\times$ and $2\times$ the value in the baseline model"

7a. l. 195ff "The population of functional parameter combinations enabled us to calculate the Pearson's correlation coefficient r for all pairs of conductance density parameters. We found weak pairwise correlations indicating low dependencies between each pair of channels and thus increasing the robustness of the model (Figure S5).": when you say "functional parameter combinations", do you mean the subset of GC models with valid parameter combinations? Please use consistent terminology.

Our response 23: Yes, we meant the subset of GC models with valid parameter combinations. Accordingly, we changed the wording in the following way to avoid confusion:
"The population of GC models with valid parameter combinations enabled us to calculate the Pearson's correlation coefficient r for all pairs of conductance density parameters. We found weak pairwise correlations indicating low dependencies between each pair of channels and thus increasing the robustness of the model (Figure S5)."

7b. l. 208 "In our selection of random parameter combinations, we found suitable models covering the": suitable models → valid models? Please check for other instances in the manuscript where 'valid' should be used for consistency of terminology.

Our response 24: Thank you for the comment. Throughout the manuscript, we have changed the wording to keep the terminology consistent.

8. Figs. 3C and 3D, top right: the blue asterisks appear to be positioned too far to the left to denote the mean of the distribution.

Our response 25: Thanks, we have fixed it.

9. l. 466 "Similar to Olypher and Calabrese (2007) and Achard and De Schutter (2006) we showed that": shouldn't the order of the citations be reversed based on either temporal or alphabetic order?

Our response 26: Thank you, we changed the order of the citations accordingly.

10. l. 529f "in which case they will most likely affect our general understanding of how evolution deals with complex organisms.": the last sentence makes a good point, but could be improved; you could change to something like "[..] how evolution deals with complex dynamics (Koch and Laurent, 1999 [Science 284:96–98])".

Our response 27: Thank you. We changed the phrasing in the following way:
"It will be interesting to investigate whether our findings here translate to other biologically complex systems, in which case they will most likely affect our general understanding of how evolution deals with complex dynamics (Koch and Laurent, 1999)"

11. l. 706 "Druckmann S (2007)": several authors missing

Our response 28: Thank you, we changed the reference accordingly.

12. l. 727 "Gunay C, Edgerton JR, Jaeger D (2008)": Gunay → Günay

Our response 29: Thank you for noticing this typo. We have corrected it in the manuscript.
